# Integrating within-species variation in thermal physiology into climate change ecology

**DOI:** 10.1098/rstb.2018.0550

**Published:** 2019-06-17

**Authors:** Scott Bennett, Carlos M. Duarte, Núria Marbà, Thomas Wernberg

**Affiliations:** 1 Global Change Research Group, Institut Mediterrani d'Estudis Avançats (CSIC-UIB), Miquel Marquès 21, 07190 Esporles, Spain; 2 King Abdullah University of Science and Technology (KAUST), Red Sea Research Center (RSRC) and Computational Bioscience Research Center (CBRC), Thuwal 23955-6900, Saudi Arabia; 3 School of Biological Sciences, UWA Oceans Institute, University of Western Australia, Cnr Fairway and Service Road 4, Crawley, WA 6009, Australia

**Keywords:** climate variability, local adaptation, phenotypic plasticity, acclimation, niche conservatism, thermal safety margins

## Abstract

Accurately forecasting the response of global biota to warming is a fundamental challenge for ecology in the Anthropocene. Within-species variation in thermal sensitivity, caused by phenotypic plasticity and local adaptation of thermal limits, is often overlooked in assessments of species responses to warming. Despite this, implicit assumptions of thermal niche conservatism or adaptation and plasticity at the species level permeate the literature with potentially important implications for predictions of warming impacts at the population level. Here we review how these attributes interact with the spatial and temporal context of ocean warming to influence the vulnerability of marine organisms. We identify a broad spectrum of thermal sensitivities among marine organisms, particularly in central and cool-edge populations of species distributions. These are characterized by generally low sensitivity in organisms with conserved thermal niches, to high sensitivity for organisms with locally adapted thermal niches. Important differences in thermal sensitivity among marine taxa suggest that warming could adversely affect benthic primary producers sooner than less vulnerable higher trophic groups. Embracing the spatial, temporal and biological context of within-species variation in thermal physiology helps explain observed impacts of ocean warming and can improve forecasts of climate change vulnerability in marine systems.

This article is part of the theme issue ‘Physiological diversity, biodiversity patterns and global climate change: testing key hypotheses involving temperature and oxygen’.

## 1. Introduction

Contemporary rates of climatic warming are profoundly changing species distributions [1,2] and the structure and function of ecological communities across many parts of the globe [3,4]. This demands a robust framework to forecast the impacts of warming on species. Thermal sensitivity (*S*) of an organism is defined here as the proximity (in °C) between the upper (*T*_max_) and lower (*T*_min_) limits of an individual's fundamental thermal niche, and the maximum (*H*_max_) and minimum (*H*_min_) ambient temperature extremes it experiences in its local habitat, respectively. That is, *S*_max_ = *T*_max_ − *H*_max_ and *S*_min_ = *T*_min_ − *H*_min_. Warming vulnerability (*V*), in turn, is a function of exposure (i.e. rate of warming, *E*) and inherent sensitivity to increasing temperatures (*V* = *S*/*E*) and describes the number of years before local temperatures are projected to surpass *T*_max_ in a given location [5]. As such, thermal sensitivity and vulnerability are dynamic as they integrate intrinsic differences in the thermal niche within and between species and extrinsic differences in climatology between locations over multiple temporal scales (i.e. weeks to decades).

Extensive experimental work has uncovered the mechanisms underpinning how temperature shapes and limits physiological performance, from genomic and biochemical processes to cellular and whole organism function (reviewed in [6–8]). The shapes of thermal performance windows display remarkable similarity across taxa [6,9] and have helped build a strong understanding of how climate change impacts the physiology and ecology of plants and animals [6,7,10,11]. At the same time, variation in the breadth and absolute limits of thermal niches across space and time generates an enormous diversity of potential thermal sensitivity scenarios among individuals within a species range. The aggregate effects of individual variation in thermal performance can have important implications for population-level vulnerability to warming [12] and short-term extreme events (e.g. marine heatwaves [13]). Yet, when applied to broad spatial scales [14] or multi-species assessments of warming vulnerability [15], within-species variation in thermal physiology is often overlooked [16], and instead a single ‘species-level’ thermal niche is often inferred from realized thermal distributions [15,17], or empirical measurements of the fundamental thermal niche from a single experimental population [14]. Thus, implicit assumptions about how species' thermal niches may differ (or not) at the population level are often made despite remaining largely untested.

The aim of this review is, therefore, to look at the effects of within-species variation in thermal limits on the sensitivity of marine organisms. Phenotypic plasticity of individuals (i.e. the ability of an individual genotype to produce different phenotypes in response to the environment) and local adaptation within a population (i.e. selection on genetic variation causing a shift of the average phenotype within a population toward a local optimum) both cause within-species variation in thermal limits at different spatio-temporal scales. We integrate local adaptation and phenotypic plasticity into current ecological theory to address the implications that assumptions about population-level variation in the thermal niche of a species can have for warming vulnerability [12]. Specifically, using the available literature, we synthesize how thermal sensitivity varies across marine species’ distributions and identify broad-taxonomic differences in the thermal sensitivity and adaptive capacity between phyla. We use these taxonomic differences to highlight the spectrum of thermal sensitivities that can occur *between-species* within marine communities and discuss the spatial, temporal and biological context under which *within-species* variation in thermal physiology may manifest and influence an individual and population vulnerability to climate warming.

## 2. Assumptions and emergent patterns in thermal sensitivity

The dominant emphasis on broad-scale species-level patterns in thermal sensitivity (e.g. [14,18–20]) has meant that macro-physiological research has often overlooked variability within species [12,13,21]. Species-level studies tend to rely on the thermal tolerance profiles from a single case study per species, usually corresponding to individuals from a single population and location. When single measures of a species' thermal limits are related to either local environmental temperatures or the global distribution of a species, implicit assumptions are being made about the thermal niche of that species, which can be broadly classified into two groups: (1) studies that link single measures of the fundamental thermal niche to a species distribution (i.e. realized thermal niche) often tacitly imply thermal niche conservatism among intraspecific populations (e.g. [14]). (2) Studies that link the fundamental niche of an experimental population to temperature metrics at the collection site often infer local adaptation, when local thermal regimes differ across a species' geographical range (e.g. [20,22]).

Use of the fundamental niche of an experimental population to infer niche filling at the species level involves the assumption that the thermal niche of the experimental population is conserved across individuals in central and marginal populations. Such thermal niche conservatism has been observed empirically for several marine species [23,24], and may be expected for highly mobile, widely dispersing or migratory species with high inter-population connectivity, or recently established populations in new thermal environments [25] (table 1). Importantly, niche conservatism underpins prevailing methods used to understand climate change impacts on species distributions (i.e. species distribution models, bioclimatic envelope models, ecological niche models) [28]. Nevertheless, many species can display marked population-level differences in thermal tolerance limits [13,29–31]. Studies that link the fundamental niche with absolute temperatures or temperature variability in a given location also tend to make species-level inferences about thermal sensitivity, based on case studies of single populations [19,20]. In doing so, these studies make the implicit assumption that individuals from other (untested) populations of the same species will be locally adapted or acclimatized. Temperature regimes can vary considerably across a species' geographical distribution in terms of seasonal variability and/or temperature extremes (see §4, below). Under such circumstances, in order for the relationship between a populations' thermal sensitivity and local climatic conditions to hold, a necessary assumption is that the thermal niche of the majority of individuals in different populations changes as a function of changes in thermal regimes throughout a species range. That is, populations living under different thermal regimes are assumed to be adapted or acclimatized to their local climate.

**Table 1.** Hypotheses relating to the influence of biotic and abiotic traits on the prevalence of locally adapted and conserved thermal niches between populations.

trait type	trait	hypothesis for thermal niche	
		local adaptation/acclimatization	niche conservatism
biotic	reproductive mode	species with low dispersive reproductive modes (e.g. brooders) will have limited gene flow among populations and high adaptive divergence *or alternatively* in species with dispersive reproductive modes but where selection pressure exceeds the homogenizing effect of gene flow, local adaptation may occur (e.g. microgeographical adaptation [26])	species with more dispersive reproductive modes (planktotrophic and lecithotrophic larvae) will have higher gene flow and decrease adaptive divergence among populations [12]
	adult motility	species with low mobility (e.g. sessile and sedentary species) will have high selection pressure from ambient environment, leading to adaptive divergence among populations	species with high mobility and broad home ranges (e.g pelagic fishes) may form geographically large populations, track optimal thermal conditions and will display low heterogeneity in thermal niche among populations [27]
	latitudinal range size/thermal niche breadth	species with broad latitudinal ranges will be exposed to a diversity of local climatic regimes, resulting in selection pressure on local populations to adapt. These populations will have a broad ‘potential’ thermal sensitivity spectrum, enabling populations to modify their thermal limits toward the species-specific limit, if the pace of warming permits	populations of species with narrow latitudinal and thermal ranges will be exposed to similar climatic conditions throughout their range and have low selection pressure to adapt to different thermal conditions. These populations will have a narrow thermal spectrum and high sensitivity to warming populations of species with broad latitudinal ranges evolved as climate generalists enabling them to occupy diverse climatic regimes and have the latent capacity to deal with diverse climatic conditions
abiotic	climatic history (evolutionary time scales)	species that have evolved under stable climates over geological time scales (e.g. SW Australia) may have more specialized thermal niches and greater propensity toward local adaptation	species that have evolved under dynamic, disruptive climates over geological time scales may have more generalist thermal niches (e.g. northern Europe)
	disturbance history (demographic time scales)	stable climatic disturbance history may promote low plasticity and greater variation in thermal limits among populations	frequent disturbance history over demographic time scales (i.e. to inherit maternal conditioning effects) may lead to greater phenotypic plasticity, broader thermal niches and therefore less population-level variation
	barriers to dispersal	barriers to dispersal will lead to genetic isolation of populations and selection pressure for genotypes suited to local conditions	

**Figure 1.**
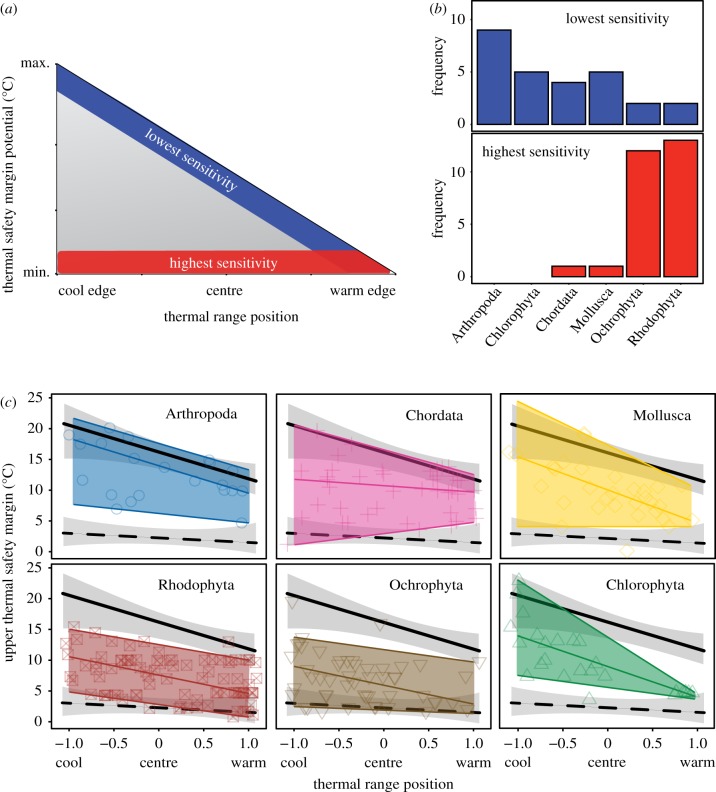
Variation in potential and observed thermal safety margins (TSMs) of populations with respect to thermal range position. (*a*) Wedge of potential TSMs for organisms with respect to thermal range position. TSMs of all organisms, irrespective of range position or niche type, occur within blue-, red- or grey-shaded areas of the triangle. Blue shading represents the axis of lowest potential sensitivity, where species-specific thermal limits are, on average, conserved across a species range. TSMs therefore decline between the cool and warm edge of a species distribution. Red shading represents the axis of highest potential sensitivity, where upper thermal limits resemble maximum local environmental temperatures (e.g. via local adaptation or phenotypic plasticity). TSMs are therefore close to zero, irrespective of range position. (*b*) Frequency of experimental populations from six marine phyla with mean upper TSMs in the upper 90th percentile (i.e. lowest sensitivity) and lower 10th percentile (i.e. highest sensitivity) portion of the observed TSM wedge. Sample sizes are: Arthropoda *n* = 21, Chlorophyta *n* = 25, Chordata *n* = 42, Mollusca *n* = 31, Rhodophyta *n* = 91, Ochrophyta *n* = 51. (*c*) Empirical relationships between TSMs and global range position of marine organisms from six marine phyla, based on upper thermal limits extracted from GlobTherm [32]. Coloured shapes illustrate TSMs from experimental populations and shaded areas represent their empirical wedge. Upper, middle and lower coloured lines represent 95th percentile, 50th percentile and 5th percentiles of points from each phylum. Solid and dashed black lines indicate overall significant and non-significant relationships of the 95th and 5th quantiles, respectively, across all marine taxa combined. Grey-shaded areas indicate the overall 95% CI of the quantile regressions, across all taxa. Individual plot of Echinodermata (*n* = 4) is not presented owing to the small sample size.

In reality, thermal limits occur across a spectrum between the maximum environmental temperature individuals experience locally and beyond the maximum temperatures a species experiences globally (i.e. species-specific limit). That is, individuals from an established population should at least survive the environmental conditions typical of that location, but may also be able to survive in much warmer conditions. Examined across a species' geographical range, this spectrum of thermal limits creates a wedge of potential thermal safety margins (TSMs) that individuals may experience (figure 1). The upper margin of the wedge represents the axis of lowest sensitivity (i.e. maximum potential TSMs), where upper thermal limits are conserved, resulting in declining TSMs between the cool-edge and warm-edge of a species' distribution, as environmental temperatures approach the species-specific thermal limit. The bottom of the wedge represents the axis of highest thermal sensitivity (i.e. minimum potential TSMs), where upper thermal limits are similar to maximum local environmental temperatures, irrespective of thermal range position. While locally adapted and acclimatized individuals are not confined to this axis, individuals and populations observed along this axis are likely to be highly adapted or acclimatized to their local thermal environment (see §3, below). Historically, the upper axis of the sensitivity wedge has been predominantly used to forecast local extirpation and species redistributions from climate change. But several studies have now highlighted how short-term warming can cause large-scale, catastrophic mortality events at temperatures well below the species-specific limit, particularly throughout central and cool-edge locations of a species distribution [13,33], consistent with the thermal sensitivity wedge.

To examine whether empirically derived TSMs do indeed form a wedge with respect to thermal range position, upper TSMs of marine organisms were quantified using GlobTherm, a global database of species' upper thermal limits [32]. To characterize the variability in upper TSM of marine organisms, we compared the mean upper thermal limit of experimental individuals from 265 marine taxa with the relative range position and local climatology of the studied organisms (methods described in electronic supplementary material).

Overall, empirical TSMs of 265 marine species from seven phyla displayed a characteristic wedge structure with respect to thermal range position, consistent with the theoretical wedge (figure 1*c*). Combining all taxa, TSMs (°C) in the upper 95th percentile of the wedge declined significantly towards the warm edge of the species range (RI, °C), as described by the fitted linear regression equation

$$\mathrm{TS}M_{\;p95\;}=-4.25\;\times \;\mathrm{RI}+16.01\;(t_{1,264}=-5.22,p<0.001).$$

Upper TSMs along the bottom 5th percentile of the wedge displayed no significant relationship with range position (*t*_1,264_ = −1.14, *p* = 0.14), consistent with expectations of local adaptation. Closer examination of the taxa contributing to the realized TSM wedge revealed taxonomic differentiation between phyla in the upper and lower portions of the wedge (figure 1*b*). Red and brown seaweeds (Rhodophyta and Ochrophyta) characterized the most sensitive axis of the wedge, accounting for 92% of observations in the bottom 10th percentile of the distribution (figure 1*c*). Twenty-three and 14% of total observations of brown and red seaweeds, respectively, were found along the high sensitivity axis, compared with 4 and 2% along the low sensitivity axis. By contrast, invertebrates (Arthropoda and Mollusca) accounted for 51% of observations in the upper 90th percentile of the distribution, while green seaweed (Chlorophyta) and vertebrates (Chordata) accounted for 18 and 14%, respectively. Forty-two per cent of total observations of Arthropoda were found along the low sensitivity axis of the wedge. All phyla displayed a broad vertical spread of TSMs consistent with the idea that marine organisms exhibit differing degrees of adaptive capacity, rather than a binary risk (figure 1*c*). The realized thermal niche breadth of species within the six main phyla ranged between 21 ± 8 and 29 ± 5°C (mean ± s.d.) with no overall difference between seaweeds and invertebrates or vertebrates.

Our analysis is based on the upper thermal limits from a single experimental population from multiple species, and therefore suggests broad-taxonomic patterns in adaptive capacity between species, rather than specific case studies of local adaptation within species. Thermal limits from multiple populations of each species would need to be tested to validate these patterns and disentangle the environmental drivers of thermal sensitivity. Nevertheless, the empirical TSM wedge generates interesting hypotheses about the potential mismatches in adaptive capacity between different taxonomic groups, and highlights the wedge of thermal sensitivity species may experience. In the following sections, we will discuss the potential biological drivers of these patterns and how spatial and temporal context influences population-level patterns of thermal sensitivity.

## 3. Biological context of thermal sensitivity

Our analysis suggests that taxonomic differences in thermal sensitivity may exist within marine communities, particularly between primary producers and higher trophic levels, which correspond to a spectrum of thermal adaptive capacity. Indeed, the high frequency of benthic seaweeds in the locally adapted portion of the wedge is consistent with a previous review that found 90% of studies on marine macrophytes displayed population-level variation in upper thermal limits [34]. Sensitivity spectrums can vary in breadth between species depending on their evolutionary and ecological histories or biological traits. For example, species that have evolved across spatially broad or seasonally variable thermal ranges may display a steep sensitivity wedge, whereby cool-edge populations live far below their species-specific limit. By contrast, species that have evolved under a narrow thermal range (e.g. Antarctic ectotherms [35]) or display high levels of local thermal adaptation (e.g. due to strong selection gradient, or low gene flow) may display a shallow sensitivity wedge, with limited capacity to absorb warming, irrespective of their range position. While no structured differences in thermal niche breadth were observed between phyla, seaweeds differ from many vertebrate and invertebrate animals in terms of biological traits (e.g mobility, dispersal) and their response to environmental drivers (e.g. light requirements), which may influence their adaptive capacity. Reproductive mode and the dispersal distance of propagules, for example, may influence population connectivity and the establishment of either locally adapted or conserved thermal niches between populations. Species with highly dispersive reproductive modes may have higher gene flow among populations, resulting in less adaptive divergence (i.e. gene swamping) and increasing the likelihood of thermal niche conservation among populations [36] (but see [26,37]). Conversely, low dispersal and limited gene flow among populations may promote the selection of genotypes that are best suited to local conditions [13]. *Scytothalia dorycarpa*, a canopy-forming seaweed from southern Australia, provides an example of this. *Scytothalia* displays strong patterns of local adaptation in its upper thermal limits along a latitudinal climate gradient [13] that is thought to be driven, in part, by limited gene flow between populations as a result of its large propagule size and a negatively buoyant thallus [38]. By contrast, many invertebrate and vertebrate species produce motile larvae with long pelagic larval phases which can facilitate gene flow, thereby helping to homogenize thermal limits between populations [39]. Long-distance dispersal can also occur in seaweeds [40] and is not ubiquitous within marine animals.

Local adaptation can also occur despite high levels of gene flow, or at small spatial scales within a species dispersal kernel. Microgeographical adaptation can arise through several mechanisms where selection exceeds the homogenizing effect of gene flow [26,37] and may be particularly relevant along steep climatic gradients or depth gradients or among proximate locations with very different climatic regimes where selection pressure on resident populations may be high and lead to variation in thermal profiles, despite close geographical proximity. For instance, the seagrass *Posidonia oceanica*, growing from near-surface to about 40 m depth in the Mediterranean, may experience maximum seasonal temperature differing by 10°C across the depth range, but similar minimum temperatures, experienced at the time of winter convective mixing [41,42]. Hence, local adaptation in this case may occur within horizontal spatial scales of hundreds of metres, and propagules from more thermally resistant genotypes in shallow waters may provide added thermal resistance to the population in deeper waters with long-term warming. Species with high dispersal will have a better capacity to infill and recover from localized disturbance events with thermally resistant genotypes from warmer regions more readily than species with poor dispersal capabilities [12]. Infilling rates could have important implications on levels of population fragmentation, localized extinctions and community stability in the face of warming, particularly for species with high rates of local adaptation in central and marginal populations [13].

Finally, while lethal or critical thermal limits may be used to describe an organism's thermal sensitivity; these metrics, by definition, represent the endpoints of a species' thermal niche and generally exceed temperatures at which other important biological processes are affected. Physiological performance typically increases from a lower thermal limit to optimal temperatures, before decaying rapidly toward an upper thermal limit. Declining performance beyond optimal temperatures can have important ecological implications for population-level viability [6]. Moreover, different biological processes can have their own thermal niche, nested within the broader thermal range described by lethal limits. Reproduction, for example, may have a lower thermal performance window than adult growth [38] and survival [43]. Identifying the thermal range of biological processes that are limiting to individual fitness and population persistence will be necessary in order to refine estimates of thermal sensitivity and enhance our ability to forecast the vulnerability of different populations to ocean warming and extreme climatic events.

## 4. Spatial variation in the thermal sensitivity

The relationship between an organism's fundamental thermal niche and the environmental temperature regime that it experiences provides a strong foundation to examine the expected spatial variability of the thermal niche [44]. The climate variability hypothesis (CVH) posits that there is a positive relationship between an organism's thermal tolerance breadth and the level of climatic variability it experiences with increasing latitude [45]. While originally proposed as an explanation for Rappaports rule [46], more recently the CVH has been used to explain the climate change vulnerability of species at different latitudes, leading to the idea that high-latitude species will be more resistant to warming owing to their broader thermal niches and larger TSM compared with tropical species [20,22]. The CVH has been tested across a range of terrestrial and aquatic organisms, including insects [47–49], bivalves [50], lizards [51–53] and amphibians [54–56]. For many terrestrial species, the mechanism driving the relationship between thermal tolerance breadth and ambient conditions arises from the conservation of upper thermal limits and labile lower limits [18,47].

In marine systems, upper and lower thermal limits are thought to be more closely coupled as a result of oxygen limitation on physiological functioning, such that a change in thermal limits at one end of a species thermal tolerance breadth is associated with a change at the other. Also, with the exception of changes in depth distribution [57], marine organisms generally have a limited ability to modify or seek refuge from their ambient thermal environment [58], arguably placing a greater selection pressure on the upper thermal limits of marine compared with terrestrial organisms. Moreover, latitudinal patterns in climatic variability are subject to major regional anomalies in the ocean, particularly in polar regions, and the temperate Southern Hemisphere, thereby calling for a re-evaluation of expectations from the CVH. For example, the pattern of increasing thermal tolerance breadth of an organism with increasing latitude does not apply to marine polar organisms, as the ambient thermal range is constrained by the freezing point of seawater (around −2°C) and the high thermal capacity of seawater (maximum temperatures in polar waters around 8°C). While many marine organisms survive under ice cover, temperatures beneath the ice remain stable, thereby reducing the scope for extreme cold hardiness as observed in some terrestrial organisms [59]. Moreover, while low-latitude tropical regions (i.e. ±10° of Equator) generally have stable climates, mid-latitudinal regions are often composed of a mosaic of thermal variabilities, with some regions displaying low variability and others high-temperature variability, particularly in coastal regions, where tidal regimes, currents, upwelling and bathymetry can all have a strong influence on local thermal regimes [60,61]. Across latitudinal clines on both the eastern and western coastlines of Australia (10–40°S), for example, seasonal climate variability remains relatively low, largely owing to strong poleward-flowing boundary currents. Also, across longitudinal clines within temperate latitudes, large differences in seasonal climate variability can be observed, particularly between the eastern (18.3 ± 3.1°C yr^−1^) and western (4.4 ± 1.5°C yr^−1^) coastlines of the United States, and between the Atlantic (5.6 ± 1.4°C yr^−1^) and Mediterranean (12.6 ± 1.0°C yr^−1^) coastlines of southern Europe. An extreme case of thermal variability is found in the Arabian Gulf, where marine organisms, which include many species shared with the Indian Ocean and Red Sea, may experience seasonal thermal ranges above 20°C within individual locations [62,63]. Therefore, while temperature variability may place a strong selection pressure on the thermal niche of marine organisms, its influence may need to be considered in a more regional, rather than latitudinal, context in the ocean. This could have strong implications for the thermal sensitivity of marine organisms, whose distributions may span across regions of high and low thermal variability.

## 5. Temporal context of thermal sensitivity

**Figure 2.**
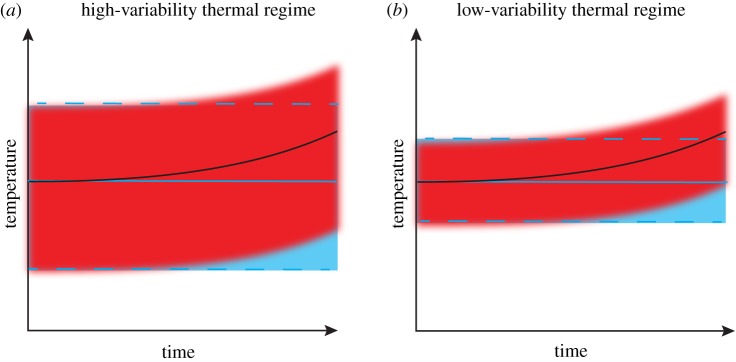
(*a*,*b*) Representation of two populations living in variable and stable thermal environments, respectively. Solid and dashed blue lines illustrate the mean and maximum and minimum temperatures over time in the absence of warming. Black solid line and red shaded area illustrate mean temperature and the thermal range over time with warming in a variable and stable thermal environment. Under equal rates of warming, mean temperatures fall within the historical range of experienced conditions (i.e. between the blue dashed lines) for organisms living in the variable climate, but exceed historical temperatures experienced by the stable climate. However, when climatic extremes are considered, future extreme temperatures will exceed historical limits in both variable and stable environments.

The temporal context in which warming is considered (e.g. long-term mean climatic warming versus short-term extreme events) also affects how temperature variability influences the sensitivity of locally adapted populations. If the thermal niche of a locally adapted population reflects the local thermal range of its habitat, then populations living in areas with a broad thermal range may be able to absorb larger increases in mean temperature than those adapted to low-variability environments (figure 2). For example, a 1°C increase in the mean will fall within the historic thermal range of a variable environment but may fall outside the historic thermal range of a stable environment (figure 2). However, in the context of extreme events, if local temperature variability remains constant under warming, then a 1°C anomaly above summer maximum may fall beyond historical temperature regimes in both stable and variable environments (figure 2). In this case, populations adapted to variable environments would need larger TSMs than those adapted to stable environments, in order to be more resistant to warming. Short-term extreme temperature anomalies (i.e. marine heatwaves) are increasing in magnitude and frequency [64] and can have devastating impacts on locally adapted or acclimatized organisms, such as corals [65,66], seagrasses [67] and seaweeds [13]. These events act as a force selecting the most resistant genotypes within a population and are, therefore, expected to play a major role in the evolution of the TSM of local populations. Future research examining changes in TSMs of locally adapted populations in response to local temperature variability is required to better understand their sensitivity to extreme events.

**Figure 3.**
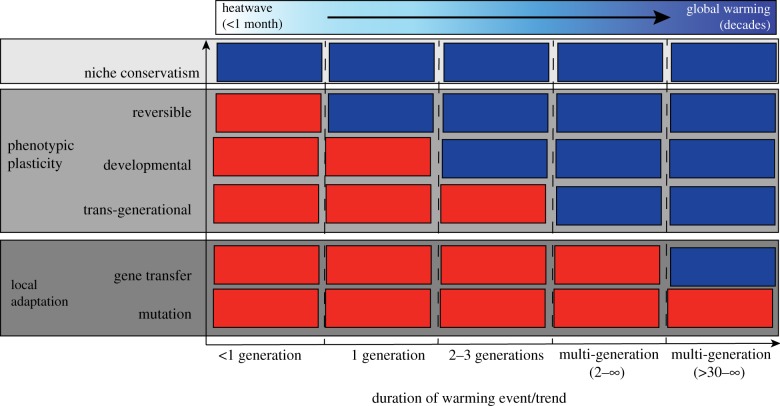
Relationship between the plastic and genetic processes that can result in phenotypic variation in thermal limits among populations, and the duration of a warming event or trend where these differences could be observed. Red boxes represent scenarios where different thermal limits may be detectable among populations in response to thermal stress. Blue boxes represent scenarios where no difference in thermal limits among populations will be detectable in response to thermal stress.

Both plastic and evolutionary processes can result in temporal variation in thermal limits and are often difficult to distinguish experimentally [68]. However, thermal sensitivity based on phenotypic plasticity or adaptation may manifest differently depending on the duration of the warming event or trend being examined [16,69]. For example, populations living under different climatic regimes may have different upper thermal limits at any point in time as a result of reversible acclimation. If exposed to an extreme short-term anomaly such as a marine heatwave, these differences could have important implications for the sensitivity of the different populations to disturbance, such as greater mortality of individuals in the cool-acclimated population, yet no observable impact on the warm-acclimated population. By contrast, under a more gradual, long-term warming trend, the cool-acclimated individuals may have time to adjust toward the optimal species-specific limit and therefore the same two populations may display a more similar upper thermal limit (figure 3). Trans-generational acclimation or evolutionary adaptation may result in a slower rate of homogenization of thermal limits between populations. Therefore, population-level differences in thermal limits may also be relevant to sensitivity over longer periods of time, including decadal warming trends, typically associated with climate change (figure 3). Three factors that can influence the rate of homogenization of thermal limits between populations are (1) acclimation time, (2) environmental warming rate and (3) the generation time of the organism.

(1) The temperature and time that an organism spends acclimating can have a strong bearing on its critical and lethal thermal limits [70]. Organisms have a maximum acclimation capacity representing the maximum amount of plastic physiological adjustment they can make (i.e. the species-limit for sub-optimal populations). Acclimation at warmer, subcritical temperatures can enable populations to increase their critical or lethal limits toward the species-limit [71]. However, the duration of time needed by organisms to fully acclimate can differ greatly between taxonomic groups and climatic regions. For example, some tropical and temperate fishes can fully acclimate to elevated temperatures within 2–5 days [72]. By contrast, some Antarctic fish species require up to 20 days to fully acclimate to warmer conditions [73], while some Antarctic invertebrates can require between two and five months [70]. Such taxonomic and regional differences could have an important influence on whether sub-optimal populations can adjust their thermal limits to short-term thermal anomalies or not.
(2) Environmental warming rates can also have a strong bearing on the upper thermal limits of locally acclimated organisms [74,75]. In thermal tolerance experiments of Antarctic ectotherms, Peck *et al*. [74] observed a negative relationship between the upper thermal limits of marine invertebrates and the rate of increase in temperature. Species exposed to a 1°C day^−1^ increase in temperature displayed a median thermal limit of 12°C, compared with a 7.5°C limit at a warming rate of 1°C week^−1^ and a 3.1°C upper thermal limit at a warming rate of 1°C month^−1^. Thus, the rate of onset and duration of warming events such as marine heatwaves are likely to have an important bearing on the upper thermal limits of organisms and their sensitivity to warming [71]. Moreover, these results suggest that high tolerance limits of local populations in response to short-term extreme warming events may not necessarily be indicative of the upper thermal limits that can be expected in response to decadal warming trends, as long-term limits may indeed be lower than expected.
(3) Species’ generation times and life-history traits can also have an important influence on sensitivity and the agility of populations to respond to warming. Sub-optimal populations of species with short generation times will have a greater capacity to undergo both plastic and genetic adjustments in their thermal niche in response to climate warming. Indeed, while genetic change to population thermal limits requires multiple generations (e.g. for thermally tolerant genotypes to be selected for within the available gene pool), species with generation times of days to weeks [76] may have the capacity to adapt more quickly than species with generation times of multiple decades, and undergo ‘more rapid’ developmental or trans-generational acclimation.

## 6. Conclusion

The complex interactions between an individual's thermal physiology and the environment mean that accurately predicting sensitivity to climate change presents significant challenges, particularly when extrapolating from local to global scales or from short-term experiments to long-term warming, and even more so when considering the diversity of life on Earth. Here we identify that, within any given community, thermal sensitivity may occur across a spectrum, ranging from organisms with highly conserved thermal niches, to others with locally adapted thermal niches. We identify that while this spectrum is implicit within the existing literature, a stark contrast in thermal sensitivity can result from having a conserved versus a locally adapted thermal niche, which is seldom recognized. Indeed, the wedge of potential thermal sensitivity helps to explain already observed patterns of population extirpation from thermal anomalies that would not have been predicted by the thermal distribution of the species alone [13,33]. Organisms with a conserved thermal niche display the lowest sensitivity to warming, particularly within central to cool-edge populations of a species' geographical range, where locally adapted individuals may display comparatively higher sensitivity. Including within-species variation in thermal physiology into climate change vulnerability predictions will require recognizing the nuanced spatial mosaics in climate heterogeneity at local and regional scales which may influence the sensitivity of locally adapted organisms, particularly in coastal marine systems. Moreover, it will require careful consideration of the temporal scale of impacts being examined and it may be necessary to move beyond a focus on critical and lethal limits *per se*, and instead identify the thermal niche of processes that best reflect long-term population viability. Recognizing the diversity of biotic responses to warming will enable us to identify hotspots and safe-spots of climate change vulnerability and better allocate resources to respond to impending impacts.

## Supplementary Material

Quantification of empirical wedge of thermal safety margins

## Data accessibility

Thermal tolerance data used in figure 1 were accessed from the GlobTherm database, freely available from the Dryad Digital Repository: https://doi.org/10.5061/dryad.1cv08 [77]. Species distribution data were obtained from the Global Biodiversity Information Facility (GBIF; www.gbif.org) and Ocean Biogeographic Information System (OBIS; www.iobis.org) online databases. All SST data were obtained from the National Center for Environmental Information (NCEI), https://www.ncdc.noaa.gov/oisst [78].
