# Supplementary material for: Integrating within-species variation in thermal physiology into climate change ecology
Source: Philos Trans R Soc Lond B Biol Sci. 2019 Jun 17;374(1778):20180550. doi: 10.1098/rstb.2018.0550 (PMC6606463; doi:10.1098/rstb.2018.0550)
Supplement: Quantification of empirical wedge of thermal safety margins [file rstb20180550supp1.docx]

Integrating within-species variation in thermal physiology into climate change ecology

Scott Bennett^1^, Carlos M. Duarte^2^, Núria Marbà^1^, Thomas Wernberg^3^

^1^ Global Change Research Group, Institut Mediterrani d’Estudis Avançats (CSIC-UIB), Miquel Marquès 21, 07190 Esporles, Spain

^2^ King Abdullah University of Science and Technology (KAUST), Red Sea Research Center (RSRC) and Computational Bioscience Research Center (CBRC), Thuwal, 23955-6900, Saudi Arabia

^3^ School of Biological Sciences & UWA Oceans Institute, University of Western Australia, Cnr Fairway and Service Road 4, Crawley 6009, WA, Australia.

Author for correspondence: Scott Bennett, email: sbennett@imedea.uib-csic.es

Supplementary material

*Quantification of empirical wedge of thermal safety margins*

To examine whether the TSMs of marine organisms conform to expectations of the potential TSM wedge, we compared fundamental thermal limits of marine organisms within the GlobTherm database (1, 2), with the relative range position of the organism being tested. GlobTherm comprises empirically measured thermal limits of more than 2000 species from terrestrial and aquatic realms (1, 2). The global distribution of marine species within the GlobTherm database were extracted from the Global Biodiversity Information Facility (GBIF) and Ocean Biogeographic Information System (OBIS) databases using the rgbif (3) and robis (4) packages, respectively, in R (R Development Core Team, 2018). Species with fewer than three observations available, including the experimental collection site, were excluded from analyses as their realised distribution was considered to be insufficiently described. 266 marine species from seven phlya met the criteria for the analysis and were included in the database.

Sea surface temperature’s (SST), based on daily SST maps with a spatial resolution of 1/4°, were obtained from the National Center for Environmental Information (NCEI, <https://www.ncdc.noaa.gov/oisst> (5)). These maps have been generated through the optimal interpolation of Advanced Very High Resolution Radiometer (AVHRR) data for the period 1981-2016. In order to characterise the thermal niche position for experimental populations of each species, mean temperatures in the site of collection were compared to mean habitat temperatures throughout each species range of origin. The lowest mean-SST across a species observed geographic range was used to characterise the lower limit of a species realised thermal niche. Similarly, the highest mean-SST from any observed cell across a species distribution was used to characterise the upper realized thermal limit. Mean SST at the experimental collection sites were then standardised into a Range Index (6), relative to the thermal range observed across a species realized distribution, using the equation: RI = 2(S_M_- D_M_)/D_B_ where S_M_ = the mean temperature at the experimental collection site, D_m_ = the thermal midpoint of the species global thermal distribution and D_B_ = The realised thermal breadth that the species experiences across its distribution. The RI scales from -1 to 1, whereby ‘-1’ represents the cool, leading edge of a species distribution, ‘0’ represents the thermal midpoint of a species distribution and ‘1’ represents the warm, trailing edge of a species distribution (6). RI enables relative range positions to be compared between species. Realised thermal niches were characterised using mean-SST’s as opposed to p01 and p99 temperatures to best reflect the distribution of experimental collection sites across a species range. The thermal safety margin (TSM) of marine organisms from the GlobTherm database were measured as the difference (in °C) between the upper fundamental thermal limit, reported in the database and the maximum summer temperature (99^th^ percentile SST between 1981-2016) from the site of collection. Local SST-range from the site of collection was characterised as the difference between minimum (p01 SST) and maximum (p99 SST) temperatures from the site of collection during the period 1981-2016.

To examine whether observed TSMs form a wedge with respect to thermal range position, upper TSMs of marine organisms were compared to the relative range position of the studied organism. We considered organisms with TSMs within the lowest 5^th^ percentile to be consistent with expectations of a locally-adapted population (i.e. upper thermal limits closely correspond to upper local environmental temperatures), making them characteristic of the lower, most sensitive extreme of the adaptive capacity spectrum. Similarly, we considered organisms with TSMs in the 95^th^ percentile to be consistent with conserved-niche populations, with the expectation that upper TSMs should decline toward the warm range edge of species’ distributions. Quantile regression splines were used to examine the relationship between TSM and range position for the 5^th^ and 95^th^ percentiles. All models were fit using the ‘quantreg’ package (7) in R (R Development Core Team, 2018). 95% confidence intervals for each spline model were calculated using the ‘boot.rq’ function.

**References**

1. Bennett JM, Calosi P, Clusella-Trullas S, Martínez B, Sunday J, Algar AC, et al. Data from: GlobTherm, a global database on thermal tolerances for aquatic and terrestrial organisms. Dryad Digital Repository2018.

2. Bennett JM, Calosi P, Clusella-Trullas S, Martínez B, Sunday J, Algar AC, et al. GlobTherm, a global database on thermal tolerances for aquatic and terrestrial organisms. Scientific data. 2018;5:180022.

3. Chamberlain S, Barve V, Mcglinn D, D O. _rgbif: Interface to

the Global Biodiversity Information Facility API_. . 1.0.2 ed. R2018.

4. Provoost P, S B. “robis: R Client to access data from the OBIS API.” Ocean Biogeographic Information System.: Intergovernmental Oceanographic Commission of UNESCO. ; 2018.

5. Reynolds RW, Smith TM, Liu C, Chelton DB, Casey KS, Schlax MG. Daily high-resolution-blended analyses for sea surface temperature. Journal of Climate. 2007;20(22):5473-96.

6. Sagarin RD, Gaines SD. Geographical abundance distributions of coastal invertebrates: using one‐dimensional ranges to test biogeographic hypotheses. Journal of Biogeography. 2002;29(8):985-97.

7. Koenker R. quantreg: Quantile Regression. R package version 5.36. ed2018.
